# Qualitative methods and patient and public involvement in trials: opportunities and pitfalls

**DOI:** 10.1186/1745-6215-16-S2-P75

**Published:** 2015-11-16

**Authors:** Pat Hoddinott, Alicia O'Cathain, Isabel Boyer, Sandy Oliver

**Affiliations:** 1 Nursing Midwifery and Allied Health professions Research Unit, University of Stirling, Stirling, UK; 2 Medical Care Research Unit, School of Health and Related Research, University of Sheffield, Sheffield, UK; 3 Lay member of the NIHR Health Technology Assessment Commissioning Board, London, UK; 4 Social Science Research Unit, UCL Institute of Education, London, UK

## 

Qualitative research and public patient involvement (PPI) in trials have increased over recent years, and can occur at many stages from inception to implementation. They offer different strengths and limitations, with both often needed to gain a real world perspective. Yet increasingly they are portrayed as a dichotomy in the way they are written about in grant applications, protocols and reports. How helpful is this?

Qualitative research methods seek a deeper understanding of patient, health professional or other relevant perspectives on health-related conditions, or services within a wider social and cultural context. A trial is conceptualised as a unique social and cultural situation, where the intervention is just one event amongst many, often with both intended and unintended consequences. PPI is a philosophy of research being shaped by the people it is undertaken for and funded by, underpinned by the World Health Organisation, *Ottawa Charter for Health Promotion 1986* and recent reforms in the UK *Health and Social Care Act 2012*. We explore this diagrammatically, to understand the contribution of each and the overlap where integration occurs.

We will present a series of fallacies drawn from an analysis of the issues that we have observed as researchers, members of funding boards and in the literature. We consider the strengths and limitations for each approach, by asking why, what, where, who, when, and how? The question of how to assist trial researchers to find the best possible approach for their research questions is considered, including whether more prescriptive guidance is indicated.

